# Targeting the hypoxia signaling pathway with nanomedicine to reverse immunotherapy resistance

**DOI:** 10.20517/cdr.2025.132

**Published:** 2025-09-02

**Authors:** Xiaoliang Cheng, Peixing Wang, Hongqiang Lyu, Yonghyun Lee, Juyoung Yoon, Haiyan Dong

**Affiliations:** ^1^Department of Pharmacy, The First Affiliated Hospital of Xi’an Jiaotong University, Xi’an 710061, Shaanxi, China.; ^2^College of Pharmacy, Ewha Womans University, Seoul 03760, South Korea.; ^3^Department of Chemistry and Nanoscience, Ewha Womans University, Seoul 03760, South Korea.; ^4^Department of Materials Science and Engineering, Yonsei University, Seoul 03722, South Korea.; ^5^School of Automation Science and Engineering, Faculty of Electronic and Information Engineering, Xi’an Jiaotong University, Xi’an 710049, Shaanxi, China.; ^6^Graduate Program in Innovative Biomaterials Convergence, Ewha Womans University, Seoul 03760, South Korea.; ^#^Authors contributed equally.

**Keywords:** Immunotherapy resistance, HIF-1α, tumor-associated macrophages, T cell exhaustion, immune evasion, vascular normalization, nanomedicine

## Abstract

Immunotherapy has emerged as a major therapeutic strategy for cancer; however, immunotherapy resistance remains a significant challenge. Hypoxia, a key hallmark of the tumor microenvironment resulting from the imbalance between the high oxygen demand of rapidly proliferating cancer cells and the limited supply from abnormal blood vessels, plays a central role in driving immunotherapy resistance. Hypoxia-inducible factor-1α (HIF-1α) and its downstream signaling pathways contribute to this resistance by promoting macrophage polarization toward the protumorigenic M2 phenotype, inducing T cell exhaustion, facilitating immune evasion, enhancing angiogenesis, and activating other resistance mechanisms. The review highlights the mechanisms by which hypoxia regulates resistance to immunotherapy and provides a comprehensive overview of nanotechnology-based strategies designed to counteract hypoxia-induced resistance. Finally, the prospects and challenges of translating nanomedicine-based drug delivery systems into clinical practice for overcoming immunotherapy resistance are outlined.

## INTRODUCTION

In recent decades, immunotherapy has emerged as a major treatment strategy for cancer, complementing traditional approaches such as chemotherapy, surgery, radiotherapy and targeted therapy. Patients now benefit from a wide range of immunotherapeutic approaches. However, despite these advances, immunotherapy still faces the profound challenge of low response rates^[1]^, with resistance representing the primary cause of treatment failure.

The tumor microenvironment (TME) is a highly complex ecosystem composed of diverse immune cells, stromal cells, fibroblasts, cytokines, chemokines, extracellular matrix components, and blood vessels^[2]^. The TME is characterized by features such as low pH, hypoxia, abnormal vasculature, elevated interstitial pressure, a dense extracellular matrix, and immunosuppression. These features not only support neoplasm initiation, progression, infiltration, and metastasis but also drive resistance to cancer therapies^[3]^. Hypoxia, a defining hallmark of the tumor milieu, has been shown to play a central role in resistance to chemotherapy and radiotherapy^[4,5]^. Increasing evidence also demonstrates that hypoxia promotes resistance to immunotherapy through multiple mechanisms^[6-8]^, making it a critical focus in efforts to overcome immunotherapy resistance. Hypoxia-inducible factor-1α (HIF-1α) and its signaling pathway are key mediators that enable cellular adaptation to hypoxic conditions, promote immunosuppression, and drive tumor progression and resistance to immunotherapy^[9]^.

Over the past few decades, nanotechnology has achieved remarkable progress, leading to the approval and clinical application of various nanodrugs, including Doxil, Abraxane, Marqibo, and Onivyde, for cancer treatment^[10]^. Nanodrugs offer multiple therapeutic advantages: they improve the solubility of hydrophobic drugs, stabilize labile agents, regulate pharmacokinetics and tissue distribution, enable both passive and active targeting through surface modification, and reduce drug resistance while minimizing toxicity^[10-12]^. Importantly, hypoxia restricts the penetration of therapeutic agents into poorly vascularized tumor regions. Nanomaterials, with their enhanced penetration and retention properties, provide a promising platform for effective drug delivery under these conditions^[13]^. This review highlights the role of the HIF-1α signaling pathway in mediating immunotherapy resistance, including its involvement in macrophage polarization toward the M2 phenotype, T cell exhaustion, immune evasion, and angiogenesis. We further discuss potential strategies to overcome resistance by targeting the HIF-1α pathway, evaluate the opportunities and challenges of nanomedicine in this context, and explore future research directions. Ultimately, we aim to provide meaningful insights and practical recommendations to advance cancer immunotherapy.

## HIF STABILITY MODULATION AND ITS ROLE IN RESISTANCE TO CANCER IMMUNOTHERAPY

Tumor cells consume large amounts of nutrients and oxygen due to their uncontrolled proliferation. Although abnormal and impaired neovasculature develops in an attempt to increase the supply of oxygen and nutrients, this supply remains insufficient to meet tumor demands^[14]^. The hypoxia-inducible factor (HIF) family, a group of transcriptional regulators, serves as a key modulator of cellular adaptation to hypoxic stress. HIF is a heterodimeric complex with a basic helix-loop-helix structure, composed of an oxygen-sensitive α subunit and a ubiquitously expressed β subunit^[15]^. To date, three α isoforms have been identified - HIF-1α, HIF-2α, and HIF-3α - which differ in tissue distribution and gene targets^[16]^. HIF-1α, a master regulator of the hypoxic response, is constitutively expressed, whereas HIF-2α is restricted to specific tissues such as hepatocytes and is exclusive to vertebrates^[17]^. HIF-3α is also present in certain cells, though its functional role remains largely unclear^[18]^. The β subunit includes HIF-1β, which is constitutively expressed in all tissues, and HIF-2β, whose expression is limited to organs such as the kidney and brain and whose role is not yet fully explored^[17,19]^.

The stability of the α subunits is tightly regulated by oxygen availability. Under normoxic conditions, the α subunits are rapidly degraded, whereas under hypoxia, they are stabilized^[20-22]^. In normoxia, prolyl hydroxylase domain (PHD) enzymes hydroxylate conserved proline residues (Pro402 and Pro564) of the α subunits, using oxygen and α-ketoglutarate as substrates^[20,23-25]^. During hydroxylation, one oxygen atom is incorporated into the prolyl residue, while the other oxidizes α-ketoglutarate, producing succinate and carbon dioxide^[17]^. The hydroxylated α subunits are then ubiquitinated at lysine residues by the von Hippel-Lindau protein, a component of the tumor suppressor E3 ubiquitin ligase complex. This complex, composed of Cullin-2, Elongin B, Elongin C, RING box protein 1, and an E2 ubiquitin-protein ligase, targets the α subunits for degradation by the 26S proteasome^[26-28]^. In contrast, hypoxia inhibits PHD activity, allowing HIF-α to accumulate and translocate into the nucleus. There, HIF-α dimerizes with HIF-β and binds to hypoxia-response elements [5′-(A/G)CGTG-3′] in promoter regions, activating transcription of genes that promote hypoxia adaptation^[29]^.

HIF plays a central role in regulating metabolism and cellular adaptation to oxygen deprivation. Under hypoxia, it induces the expression of a broad range of genes encoding proteins involved in metabolic reprogramming, angiogenesis, proliferation, apoptosis, glucose and iron transport, genomic instability, invasion and metastasis, growth factor signaling, and resistance to chemotherapy and radiotherapy. Approximately 100 HIF-dependent genes have been identified to date^[13,30]^. Hypoxia also reduces drug efficacy by limiting penetration across hypoxic gradients^[13]^. Clinically, intratumoral hypoxia is a negative prognostic factor and a major determinant of treatment failure, poor overall survival, and increased mortality^[31,32]^.

Moreover, hypoxia contributes to an immunosuppressive TME and plays a significant role in resistance to cancer immunotherapy. It promotes polarization of macrophages toward the M2 phenotype (tumor-associated macrophages, TAMs), induces T cell exhaustion, facilitates immune evasion, and enhances tumor angiogenesis, all of which support immunotherapy resistance^[33-36]^. Consequently, targeting key hypoxia-associated pathways represents a promising strategy to overcome immunotherapy resistance. Nanotechnologies designed to inhibit HIF-1α signaling as a means of reversing immunotherapy resistance are discussed in the following section.

## MECHANISM OF HYPOXIA-REGULATED RESISTANCE TO CANCER IMMUNOTHERAPY

### Hypoxia-induced macrophage polarization and the role of TAMs in resistance to immunotherapy

#### Mechanisms of hypoxia in macrophage polarization

Tissue-resident macrophages are primarily derived from erythro-myeloid progenitors in the yolk sac or fetal liver, and partly from bone marrow progenitors. These progenitors differentiate into various types of macrophages^[37]^. Macrophages are generally classified into two subtypes: classically activated macrophages (M1) and alternatively activated macrophages (M2)^[38,39]^. Interleukin-12 (IL-12), interferon-γ, bacterial lipopolysaccharide, tumor necrosis factor (TNF) and Toll-like receptor agonists induce macrophage polarization toward the M1 phenotype^[40]^. In contrast, IL, IL-5, IL-10, IL-13, colony-stimulating factor 1 (CSF-1), transforming growth factor-β1, and prostaglandin E2 promote polarization toward the M2 phenotype^[40]^. Similarly, TAMs can be divided into the proinflammatory, tumor-suppressive M1 type and the anti-inflammatory, tumor-promoting M2-like type^[38]^.

Hypoxia plays a critical role in macrophage recruitment and polarization toward the M2 phenotype. Hypoxia stimulates the production of multiple migratory factors, including vascular endothelial growth factor (VEGF), C-C motif chemokine ligand 2 (CCL2), CCL5, and CSF-1, by carcinoma and stromal cells^[41]^. These factors recruit and retain macrophages within hypoxic tumor regions^[42]^. Once recruited, macrophages are reprogrammed into an M2-like, tumor-promoting phenotype through cytokines secreted by hypoxic cells^[43,44]^. As the tumor progresses, increasing hypoxic stress reduces the release of proinflammatory cytokines such as IL-1β, TNF-α, and CCL17 by M1-skewed macrophages, thereby further promoting M2 polarization^[45]^.

Hypoxic tumor cells primarily rely on anaerobic glycolysis for energy, resulting in excessive lactic acid accumulation^[46]^. Integrating with macrophage colony-stimulating factor, metabolic byproducts of glycolysis inhibit the nuclear factor-κB (NF-κB) pathway, suppress nitric oxide (NO) and inflammation-related cytokines, and upregulate VEGF, arginase-1 (Arg-1), and other M2-associated genes^[47-49]^. Under hypoxic conditions, lactic acid strongly promotes M2-like polarization via HIF-1, Hedgehog, mammalian target of rapamycin (mTOR), and monocarboxylate transporter/HIF-1α signaling pathways^[50,51]^. Additionally, G protein-coupled receptors, which sense the acidic TME, induce inducible cAMP early repressor expression by inhibiting NF-κB signaling, further enhancing M2-like polarization of TAMs^[52,53]^. Lactate also induces histone lysine lactylation, a recently identified epigenetic modification, which upregulates M2-associated gene expression, including ARG1^[54]^. Moreover, succinate released by tumor cells activates succinate receptor 1, driving TAM education toward the M2 phenotype through the succinate receptor 1-PI3K/HIF-1α pathway^[55]^. Hypoxia-induced CXC motif chemokine ligand 12/CXC motif chemokine receptor 4 (CXCL12/CXCR4) signaling and the endoplasmic reticulum (ER) stress-associated IRE1-XBP1 pathway also promote M2-like polarization^[56,57]^. Among these diverse pathways, Toll-like receptor, CXCL12/CXCR4, and IRE1-XBP1 signaling are considered promising therapeutic targets, and inhibitors such as resiquimod, BPRCX807, and KIRA6 have been reported^[56-58]^.

Overall, tumor hypoxia plays a pivotal role in reprogramming macrophages toward the M2 phenotype, and TAMs in hypoxic tumors predominantly exhibit an M2-like rather than M1 phenotype^[59]^.

#### The role of TAMs in resistance to immunotherapy

TAMs orchestrate multiple aspects of tumor progression^[60,61]^, and recent studies have highlighted their complex roles in promoting immune evasion and resistance to immunotherapy. TAMs release various immunosuppressive cytokines, primarily IL-10 and transforming growth factor-β (TGF-β). IL-10 inhibits Th1 cell function and reduces the production of IFN-γ, IL-2, and TNF-α, thereby suppressing T cell immune responses^[62,63]^. In addition, IL-10 promotes the activation of regulatory T cells (Tregs), further exacerbating immunosuppression^[64-66]^. TGF-β suppresses T cell and natural killer (NK) cell generation and cytotoxicity, while simultaneously enhancing the differentiation and activation of Tregs^[67]^. TAMs also release chemokines such as CCL2, which recruit immunosuppressive cells including myeloid-derived suppressor cells (MDSCs) and Tregs into the TME, thereby intensifying immunosuppression^[60]^. Arg-1 secreted by TAMs depletes L-arginine, an essential metabolite for T cell function, leading to reduced T cell proliferation and impaired activity^[68]^. Indoleamine 2,3-Dioxygenase (IDO), which is upregulated in TAMs, contributes to tumor immunosuppression by degrading tryptophan and generating immunosuppressive metabolites (e.g., kynurenine and hydroxytryptophan). These metabolites inhibit T cell function while promoting Treg proliferation and activation^[69]^. Moreover, TAMs secrete cytokines such as IL-6 and CCL20 to further recruit Tregs^[70,71]^.

TAMs also play a key role in T cell exhaustion and functional inhibition. Persistent antigen-specific synaptic interactions between TAMs and CD8^+^ T cells prevent effective T cell activation and drive T cell exhaustion. Exhausted T cells, in turn, release chemokines and growth factors that recruit monocytes into the tumor and promote their differentiation into TAMs. This establishes a self-enforcing positive feedback loop, which is further accelerated under hypoxic conditions^[72,73]^. The transcription factor interferon regulatory factor-8 (IRF8), expressed by TAMs, has also been identified as a mediator of T cell exhaustion by enhancing TAM antigen-presenting capacity^[74]^.

TAMs and MDSCs reinforce each other’s activity through IL-10 secretion. MDSCs regulate Treg expansion via IFN-γ and IL-10^[60]^, and they directly inhibit antigen-specific CD8^+^ T cell activation by overproducing reactive oxygen species (ROS) and nitrating tyrosines, thereby disrupting peptide-MHC binding^[75]^. Additionally, TAMs indirectly impair T cell function via MDSCs by promoting the conversion of ATP into adenosine^[76]^. Adenosine strongly suppresses antigen-specific T cell responses and the expression of cytotoxic effector molecules such as Fas ligand and perforin^[77]^, and it also inhibits NK cell function^[77]^.

**Figure 1:**
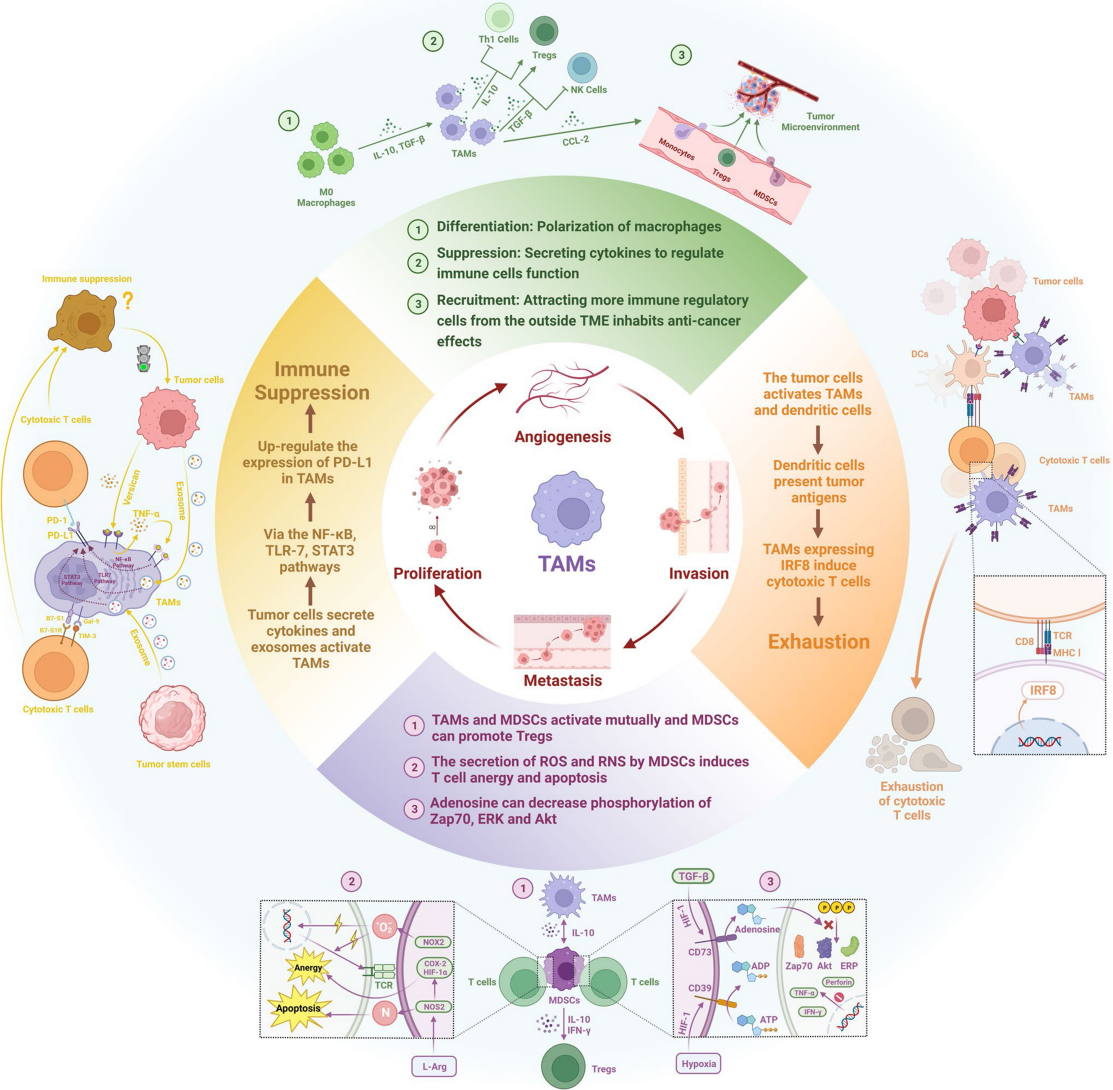
Mechanisms of TAM-mediated resistance to immunotherapy^[60]^. TAM: Tumor-associated macrophage.

TAMs express a wide range of immune checkpoint molecules, including programmed death-ligand 1 (PD-L1), PD-L2, PD-1, signal regulatory protein α (SIRP-α), and sialic acid-binding immunoglobulin-like lectin 10 (Siglec-10)^[60,78]^. Engagement of PD-1 with PD-L1 recruits Src homology 2-containing tyrosine phosphatases to the immunoreceptor tyrosine-based switch motif, suppressing downstream signaling pathways such as PI3K/Akt and Ras. This results in T cell arrest at the G1 phase, inhibition of proliferation, and enhanced conversion of naïve T cells into inducible Tregs^[79]^. TAMs have also been shown to promote T cell apoptosis through PD-L1/PD-1 signaling^[52]^. Furthermore, SIRP-α expressed on macrophages interacts with cluster of differentiation 47 (CD47), which is often overexpressed on cancer cells, delivering a “don’t eat me” signal that enables tumor immune evasion^[78]^. Similarly, the Siglec-10/CD24 axis functions as another “don’t eat me” pathway, allowing cancer cells to evade macrophage-mediated phagocytosis^[80]^. A summary of TAM-mediated mechanisms of resistance to immunotherapy is demonstrated in Figure 1.

### Regulatory mechanisms of hypoxia in T cell exhaustion

Immune checkpoint inhibitors rely heavily on the activation of T cells^[81]^. However, T cells exposed to persistent antigenic stimulation in cancer gradually become dysfunctional, a state referred to as exhaustion. Exhausted T cells are characterized by impaired effector functions, loss of memory T cell properties, and expression of various inhibitory receptors^[82]^. Hypoxia impairs the function of tumor-infiltrating lymphocytes (TILs) by disrupting mitochondrial respiration due to low O_2_ availability. Specifically, hypoxia alters electron transport within mitochondria, causing electron transfer from complex V to complex I and generating ROS, such as superoxide, which drive TIL exhaustion^[83]^. Impaired mitochondrial respiration also promotes T cell exhaustion through HIF-1α-mediated glycolytic reprogramming^[84]^. In addition, the hypoxic microenvironment triggers a transcriptional response in T cells, shifting their metabolism toward anaerobic glycolysis, alters fatty acid metabolism, and other metabolic pathways^[83,85]^. Together, nutrient and oxygen deprivation compromises T cell effector functions and exacerbates exhaustion. Chronic antigen stimulation can activate PD-1 signaling, which contributes to CD8^+^ T cell exhaustion. This state is associated with increased lipid uptake and fatty acid oxidation^[86]^. Under hypoxic conditions, the PD-1 pathway suppresses glycolysis while promoting fatty acid oxidation in T cells, further driving exhaustion^[87]^.

Hypoxia also induces ER stress by disrupting protein-folding homeostasis. This occurs because O_2_-dependent disulfide bond formation during posttranslational folding or isomerization in the ER is impaired, leading to activation of the unfolded protein response (UPR)^[88]^. Hypoxia enhances the expression of key regulators of the UPR, including binding immunoglobulin protein (BiP) and C/EBP homologous protein (CHOP)^[89]^. Activation of the UPR promotes T cell exhaustion by increasing the expression of immune checkpoint molecules on T cells [e.g., PD-1, TIM-3, lymphocyte activation gene 3 (LAG-3), and cytotoxic T lymphocyte-associated antigen 4 (CTLA-4)] as well as their ligands^[89]^.

Moreover, hypoxia suppresses T cell function by suppressing immune effector gene expression. HIF-1α interacts with histone deacetylase 1 and, in conjunction with polycomb repressive complex 2, induces chromatin remolding that epigenetically silences effector genes, leading to immune dysfunction. Targeting HIF-1α and its associated epigenetic signaling has the potential to restore T cell function and overcome resistance to PD-1 therapy^[90]^.

### Regulation mechanisms of hypoxia in immune escape

**Figure 2:**
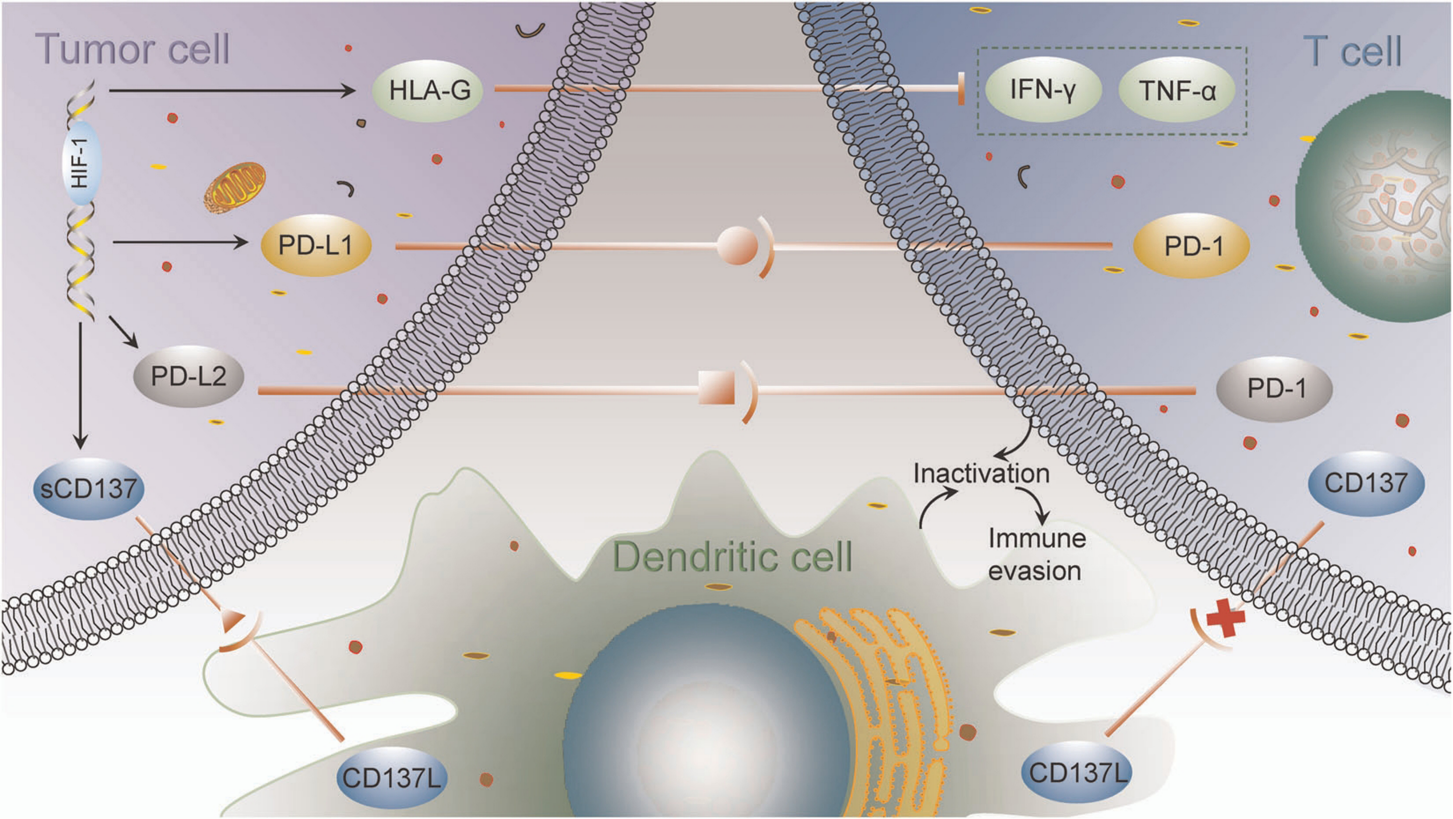
Mechanisms by which HIF-1 regulates immune checkpoint molecules and immune evasion [reproduced with permission from You *et al.* (2021)^[98]^. Copyright 2020 John Wiley and Sons]. HIF-1: Hypoxia-inducible factor-1.

Expression of various immune checkpoint proteins is a key mechanism through which carcinoma cells realize immune escape. These checkpoints have emerged as important targets for cancer therapy, with PD-L1/PD-1 representing the primary negative mediators that inhibit the anticancer activity of effector T cells. However, the therapeutic efficacy of PD-L1/PD-1 blockade has been limited, with response rates of only approximately 10%-30%^[91,92]^. Hypoxia upregulates PD-L1 expression on both carcinoma and stromal cells, while its receptors PD-1, CTLA-4, and LAG-3 are expressed on immune cells^[93]^. Binding of PD-L1 to these receptors activates intracellular signaling pathways that suppress T cell activity, leading to T cell exhaustion and tumor immune evasion^[94]^. The underlying mechanisms by which HIF-1 regulates immune checkpoint molecules and immune evasion are illustrated in Figure 2.

Hypoxia induces PD-L1 expression on MDSCs, TAMs, and dendritic cells, as well as PD-1 expression on CD8^+^ T cells^[94]^. HIF-1α elevates PD-L1 expression by directly binding to hypoxia response element 4 in the PD-L1 proximal promoter^[95]^. Hypoxia also triggers an integrated stress response that promotes phosphorylation of eukaryotic translation initiation factor 5B (eIF5B), increasing PD-L1 translation and further suppressing CD8^+^ T cells^[96]^. Inhibition of HIF-1α downregulates PD-L1 expression and enhances immunotherapy efficacy^[97]^. PD-L2, another PD-1 ligand, is also upregulated by HIF-1, and its integration with PD-1 contributes to tumor evasion of adaptive immunity^[98]^.

The immune checkpoint molecule human leukocyte antigen G (HLA-G) also mediates tumor immune escape. HIF-1 augments HLA-G transcription via hypoxia regulatory elements located in the promoter region and exon 2, with regulation being cell type-dependent^[99]^. HLA-G interacts with immunoglobulin-like transcript 2/4 and killer cell immunoglobulin-like receptor 2DL4, inhibiting cytotoxic T cells and NK cells, while activating immunosuppressive cells such as Tregs and MDSCs, thereby creating an immunosuppressive microenvironment that facilitates tumor immune evasion^[100]^.

CD137 (4-1BB), a costimulatory receptor in the TNF receptor family, is expressed on T cells. Interaction between CD137 and its ligand CD137L promotes dendritic cell, B cell, and macrophage recognition and lysis of tumor cells^[98]^. In lymphocytes, CD137 signaling enhances proliferation and effector function while preventing apoptosis, and increases antibody-dependent cytotoxicity of activated NK cells^[101]^. HIF-1α triggers transcription of soluble CD137 in neoplastic cells. Soluble CD137 binds CD137L on dendritic cells, preventing the activation of CD137-expressing T cells and thereby contributing to immune evasion^[98]^.

Several signaling pathways contribute to hypoxia-induced checkpoint upregulation, including signal transducer and activator of transcription 3 (STAT3), NF-κB, TGF-β, and elf5B. Under hypoxia, pyruvate kinase M2 phosphorylates STAT3, and loss of von Hippel-Lindau protein further activates it. Activated STAT3 inhibits HIF-1α degradation and promotes its biosynthesis^[102]^. In the hypoxic microenvironment, phosphorylated STAT3 interacts with PD-L1, facilitating its nuclear translocation. Nuclear PD-L1 acts as a transcriptional mediator, upregulating immunosuppressive molecules such as PD-L1 and PD-L2^[103,104]^. NF-κB is also activated under hypoxia, leading to enhanced expression of PD-L1 and other immune checkpoint molecules through regulation of proinflammatory cytokines such as TNF-α and IL-6^[105]^. Both STAT3 and NF-κB can directly bind the PD-L1 promoter to induce transcription^[106]^. Hypoxia amplifies TGF-β activity, which promotes PD-L1 expression via PI3K/AKT-mediated suppression of glycogen synthase kinase 3β, reducing PD-L1 degradation^[107]^. eIF5B, enriched under hypoxia, enhances PD-L1 mRNA translation^[108,109]^. HIF-1 also amplifies HLA-G transcription and protein synthesis via hypoxia regulatory elements in the HLA-G promoter and exon 2^[99]^. It directly binds genes encoding CD137 or indirectly upregulates CD137 via CD3/CD28 signaling^[110,111]^. Hypoxia induces a soluble form of CD137 that predominates over membrane-bound forms in tumor cells, blocking CD137L-mediated T cell costimulation^[101]^.

### Regulatory mechanisms of hypoxia-induced angiogenesis in immune suppression

HIF-1α acts as a transcription factor promoting tumor angiogenesis. The resulting morphologically abnormal blood vessels impair oxygen delivery and exacerbate tumor hypoxia, creating a vicious cycle. Moreover, these disordered tumor vasculatures contribute to resistance to immunotherapy^[36]^.

Malformed neovessels in tumors disrupt anticancer immune responses at multiple levels and reduce the efficacy of immunotherapy. Abnormal tumor vasculature impairs the adhesion of immune cells to endothelial cells and forms a barrier that restricts immune cell infiltration into the tumor. Endothelial cells further inhibit immune cell adhesion through intracellular sequestration or by suppressing the transcription of endothelial adhesion molecules (EAMs)^[112]^. Vascular-associated factors - including pro-angiogenic factors, inflammatory cytokines, and chemokines - downregulate EAM expression in tumor-associated endothelial cells, impairing interactions between T cells and the endothelium. TNF-α and IL-1β activate endothelial cells to initiate immune cell adhesion^[113,114]^, while bFGF and VEGF counteract proinflammatory cytokine-induced adhesion by downregulating intercellular adhesion molecule 1 (ICAM-1), vascular cell adhesion molecule-1 (VCAM-1), and E-selectin^[115]^. VEGF-A also upregulates the death mediator FAS ligand (FASL), which selectively eliminates effector T cells but spares Tregs, resulting in reduced intratumoral CD8^+^ T cells and an abundance of Tregs^[116]^. Additionally, tumor-associated endothelial cells can overexpress inhibitory molecules such as galectin-1 and endothelin B receptor, further blocking immune cell infiltration. Galectin-1 induces T cell apoptosis, and its high expression correlates with reduced T lymphocyte recruitment^[117]^. Endothelin B receptor maintains vascular homeostasis, and its integration with endothelin-1 disrupts T cell-endothelial adhesion, thereby limiting T cell infiltration^[118]^.

Hypoxia also upregulates PD-L1 expression on tumor endothelium, causing T cells to become functionally anergic within the tumor vascular lumen before entering the TME^[119]^. Hypoxia enhances IDO activity in endothelial cells, leading to tryptophan catabolism into immunosuppressive metabolites and promoting the generation of FoxP3^+^ Tregs and tolerogenic dendritic cells through aryl hydrocarbon receptor signaling^[120,121]^. Hypoxia-induced VEGF functions as a potent immunosuppressive cytokine. VEGF binding to VEGFR1 inhibits dendritic cell maturation by suppressing NF-κB signaling, thereby impairing T cell priming^[122]^. VEGF also inhibits T cell function by promoting the accumulation and proliferation of MDSCs, which in turn induce M2 macrophages and Tregs through IL-10 and IFN-γ secretion, reduce cell adhesion factor expression, interfere with immune cell extravasation, and deplete L-arginine and cystine^[123]^. Tox, a high mobility group box transcription factor, serves as a key transcriptional and epigenetic regulator of exhausted CD8^+^ T cells^[124]^. VEGF-A triggers TOX expression, driving transcriptional reprogramming toward an exhausted T cell phenotype and upregulating checkpoint inhibitor receptors; combined inhibition of PD-1 and VEGF-A can reverse resistance to PD-1 blockade^[125]^.

**Figure 3:**
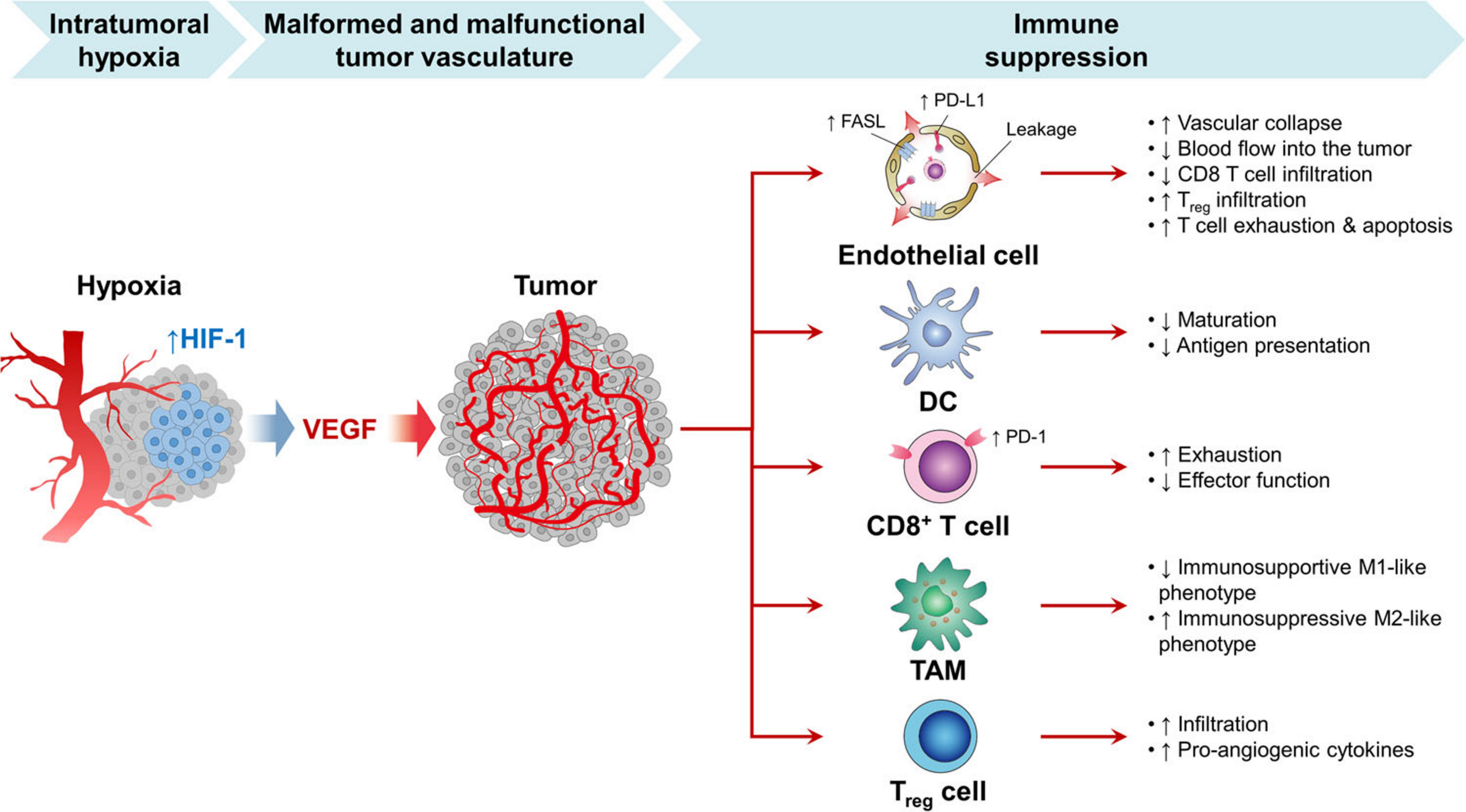
Regulatory mechanisms of hypoxia-induced angiogenesis in immune suppression^[122]^.

Beyond VEGF, other pro-angiogenic factors also contribute to immune suppression. Angiopoietin-2 recruits M2-like TAMs and Tie-2-expressing monocytes/macrophages, which enhance Treg infiltration via IL-10 and inhibit cytotoxic T cell activation^[126]^. TGF-β suppresses tumor immunosurveillance by inhibiting the functions of NK cells and T cells^[127,128]^. Placental growth factor (PlGF), a member of the VEGF family, promotes M2 polarization of TAMs^[129]^. Abnormal tumor vessels further exacerbate hypoxia, which in turn increases the secretion of chemotactic cytokines - including CCL2, CCL22, CCL28, CXCL8, and CXCL12 - that recruit immunosuppressive MDSCs, M2-like TAMs, and Tregs into the tumor^[130,131]^. The regulatory mechanisms of hypoxia-induced angiogenesis in immune suppression are summarized in Figure 3.

## NANOPARTICLES TARGETING THE HYPOXIA SIGNALING PATHWAY TO REVERSE IMMUNOTHERAPY RESISTANCE

### Nanomaterials for repolarizing or depleting TAMs

Given the central role of hypoxia-polarized TAMs in mediating resistance to immunotherapy, strategies that alleviate the hypoxic TME, reprogram TAMs toward the M1 phenotype, or directly deplete TAMs hold promise for overcoming immunotherapy resistance. A variety of nanoparticles designed for repolarizing or depleting TAMs are summarized in Table 1.

**Table 1:** Nanoparticles for repolarizing or depleting TAMs

**Therapeutic strategy**	**Carrier type**	**Payload**	**Materials**	**Ref.**
Alleviating hypoxic microenvironment	Liposomes	Hemoglobin	Egg phosphatidyl lipid-80, cholesterol, 1,2-distearoyl-sn-glycero-3-phosphoethanolamine-N-[methoxy(PEG)-2000], and soybean lecithin	[132]
Alleviating hypoxic microenvironment	Copper peroxide nanoparticle	Red blood cell membrane and protoporphyrin	Copper peroxide, chimeric peptide	[133]
Alleviating hypoxic microenvironment	Hybrid nanovesicles	IR780 and perfluorotributylamine	M1-like macrophage-derived exosomes, AS1411 aptamer, liposomes	[134]
Alleviating hypoxic microenvironment	Polydopamine nanoparticle	Oxygen	Dopamine, chitosan, polylysine	[135]
Alleviating hypoxic microenvironment	Self-assembly	Atovaquone, protoporphyrin IX	Atovaquone, protoporphyrin IX, TPGS, p K30	[136]
Alleviating hypoxic microenvironment	CaCO_3_ nanoparticles	Catalase	Poly (4-benzyl l-aspartate acid)-PEG, calcium carbonate	[137]
Alleviating hypoxic microenvironment	Cu_2-X_S nanoparticles	Fe^3+^ and doxorubicin	Cu_2-X_S, PEG, hyaluronic acid	[138]
Alleviating hypoxic microenvironment	Chiral nanoassembly	Chiral Zn complex, NaGdF_4_, porphyrin	NaGdF_4_ up-conversion nanoparticles, porphyrin metal-organic frameworks encapsulating chiral Zn complex and biotin	[139]
Alleviating hypoxic microenvironment	Albumin nanoplatform	MnO_2_, IR780, NLG919, and paclitaxel dimer	Albumin-MnO_2_, thioketal linker	[140]
Alleviating hypoxic microenvironment	MnO_2_ nanoparticle	MnO_2_ and sorafenib	1,2-distearoyl-sn-glycero-3-phosphoethanolamine-N-[aminoPEG2000 (DSPE-PEG2000), and DSPE-PEG2000-SP94 (SFSIIHTPILPL) peptide, poly(lactic-co-glycolic) acid, cholesterol, dioleoylphosphatidic acid, 1,2-dioleoyl-sn-glycero-3-phosphocholine, MnO_2_, TPGS	[141]
Alleviating hypoxic microenvironment	Carbon dots	Cu^2+^	Genistein, FA, and CuCl_2_	[142]
Directly repolarizing TAMs	Tannic acid nanoparticles	BPRCX807	Tannic acid, 1,2-dioleoyl-sn-glycero-3-phosphate, 1,2-dioleoyl-sn-glycero-3-phosphocholine, DSPE-PEG2000, and cholesterol	[56]
Directly repolarizing TAMs	Nanoemulsion	KIRA6	Medium-chain triglyceride, α-tocopherol, egg phosphatidyl lipid-80	[57]
Directly repolarizing TAMs	Metal-organic framework	Resiquimod and imatinib	Zeolitic imidazolate frameworks, polyvinyl pyrrolidone-modified Pt nanoparticles, DSPE-PEG-M2pep, tLyp-1-DSPE-PEG	[58]
Directly repolarizing TAMs	Coordination polymer	5-Aminosalicylic acid and ferric ions	mPEG2000-NH_2_, 5-aminosalicylic acid, ferric ions	[143]
Directly repolarizing TAMs	Nanoparticles	Semiconducting polymers PFODBT, atovaquone, and TMP195	Pluronic F127, hybrid cell membranes from 4T1 and Raw264.7 cells	[144]
Depleting TAMs	Biomimetic nano-red blood cells	Doxorubicin and oxygen	Maleimide-functionalized poly(ε-caprolactone), hemoglobin	[145]
Depleting TAMs	Liposome	Zoledronic acid and hematoporphyrin monomethyl ether	1,2-Dipalmitoyl-sn-glycero-3-phosphocholine, DSPE-mPEG2000-M2pep, cholesterol	[146]
Targeting HIF-1α	Liposome	Tanespimycin	1,2-Dioleoyl-sn-glycero-3-phosphocholine, DSPE-PEG 2000, cholesterol, DSPE-PEG-aminoethyl anisamide	[147]
Targeting HIF-1α	Micelles	Docosahexaenoic acid and carfilzomib	Fucoidan-selenylsulfide-docosahexaenoic acid	[148]

TAMs: Tumor-associated macrophages; PEG: polyethylene glycol; TPGS: D-α-Tocopherol PEG 1000 succinate; FA: folic acid.

#### Nanomaterials for alleviating the hypoxic microenvironment

**Figure 4:**
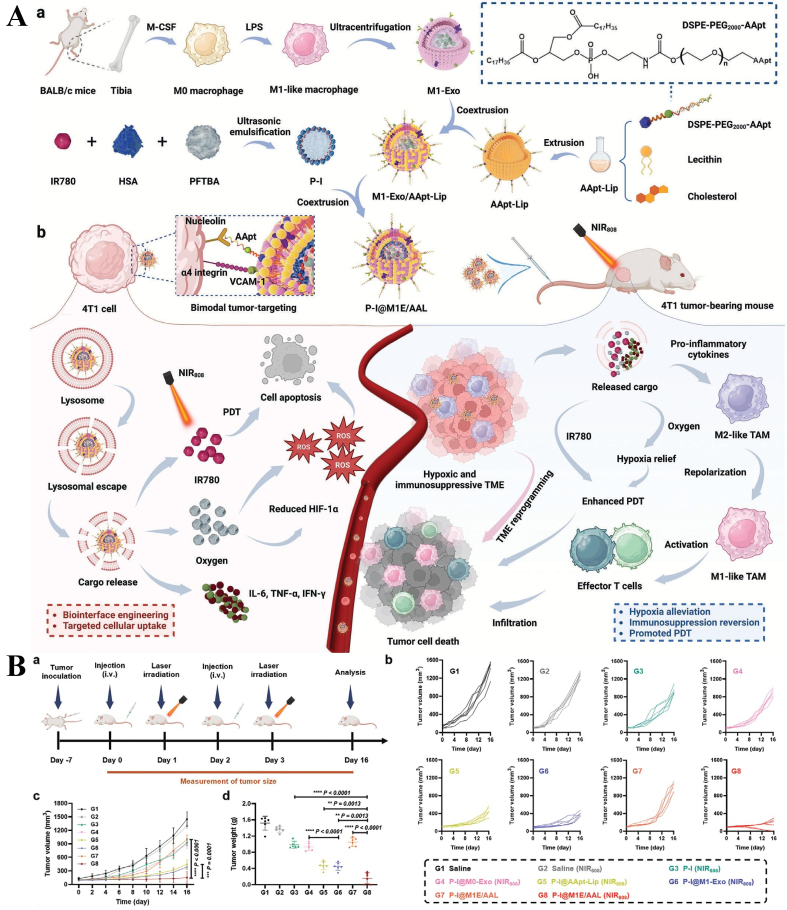
(A) Schematic illustration of the fabrication of M1-Exos and AApt-Lips hybrid nanovesicles and their regulated anticancer treatment; (B) *In vivo* antitumor effects of M1-Exos and AApt-Lips hybrid nanovesicles [reproduced with permission from Zhen *et al.* (2024)^[134]^. Copyright 2024 John Wiley and Sons]. M1-Exos: M1 macrophages-derived exosomes.

Hemoglobin-modified liposomes have been developed as promising oxygen carriers to relieve hypoxia in the TME. Treatment with these liposomes reduced the number of M2 macrophages in 4T1 tumors, resulting in a higher M1/M2 macrophage ratio. Hemoglobin-coated liposomes also significantly enhanced the efficacy of PD-1 antibody therapy by positively modulating the hypoxic milieu^[132]^. A nanoplatform composed of copper peroxide nanoparticles, which generate oxygen in acidic environments, an outer red blood cell membrane coating, and a protoporphyrin-conjugated chimeric peptide integrated into the cell membrane via electrostatic interaction was shown to reverse hypoxia through oxygen production. This platform re-educated TAMs toward the M1 phenotype and increased T lymphocyte infiltration, thereby amplifying protoporphyrin-triggered immunogenic cell death (ICD)^[133]^. Hybrid nanovesicles formed by integrating M1 macrophages-derived exosomes (M1-Exos) with AS1411 aptamer-conjugated liposomes (AApt-Lips) were employed to deliver perfluorotributylamine as an oxygen carrier and the photosensitizer IR780. These hybrid vesicles enhanced tumor photodynamic immunotherapy by repolarizing TAMs toward the M1 phenotype and promoting T lymphocyte infiltration^[134]^ [Figure 4]. Polydopamine nanoparticle-stabilized oxygen microcapsules increased oxygen concentration in hypoxic tumor regions and significantly improved the efficacy of anti-PD-1 antibody therapy against pancreatic ductal adenocarcinoma by reducing TAM infiltration and polarizing M2 macrophages to M1^[135]^.

Losartan, which can reduce solid stress and improve oxygen supply, was combined with doxorubicin liposomes to induce ICD, reprogram TAMs, and enhance PD-1 antibody efficacy. This combination successfully overcame the immunosuppressive microenvironment^[149]^. Self-assembled nanocomposites of atovaquone, protoporphyrin IX, and stabilizers mediated photodynamic ICD induction and relieved hypoxia through atovaquone-mediated inhibition of mitochondrial respiratory chain complex III, which decreased oxygen consumption and promoted TAM polarization toward the M1 phenotype^[136]^.

Catalase-loaded calcium carbonate nanoparticles induced Ca^2+^ overload, activated T cell-mediated immunity, and repolarized TAMs to the M1 phenotype by consuming protons in the TME and generating oxygen from ROS decomposition catalyzed by catalase, further boosting immune responses^[137]^. Fe-doped, doxorubicin-loaded Cu_2-x_S nanomaterials modified with hyaluronic acid and polyethylene glycol (PEG) exhibited catalase-like activity, efficiently decomposing H_2_O_2_ to produce O_2_, alleviating hypoxia, and repolarizing TAMs to M1. Combined with anti-PD-L1 nanobody therapy, these nanomaterials elicited sustained T cell responses and immune memory^[138]^. Zhang *et al.* developed a chiral nanoassembly comprising up-conversion nanoparticles and porphyrin metal-organic frameworks for the delivery of chiral Zn and biotin. This assembly facilitated H_2_O_2_ degradation into O_2_, depleted lactate, reduced HIF-1α expression, and reprogrammed TAMs, enhancing photodynamic ICD and adaptive immunity^[139]^. *In vivo*, this assembly promoted dendritic cell maturation from 11.7% in controls to 23.9%, and increased CD8^+^ and CD4^+^ T cells from 9.15% and 10.4% to 27.5% and 27.3%, respectively. A MnO_2_-based albumin nanoplatform co-loading photosensitizer IR780, indoleamine-2,3-dioxygenase 1 (IDO-1) inhibitor NLG919, and paclitaxel dimer strengthened the activity of IR780 and paclitaxel dimer via MnO_2_-catalyzed oxygen production, alleviated hypoxia, and reduced intratumoral TAMs and PD-L1 expression^[140]^. Multifunctional nanoparticles with a MnO_2_ core and a shell of lipids and poly(lactic-co-glycolic acid) for sorafenib delivery alleviated hypoxia via MnO_2_-mediated H_2_O_2_ decomposition, reduced TAM infiltration, and promoted macrophage polarization to M1, enhancing the efficacy of PD-1 antibody therapy and whole-cell cancer vaccines^[141]^. Cu-based carbon dots targeted tumors to induce ferroptosis and ICD, and O_2_ produced via Fenton-like decomposition of H_2_O_2_ mitigated hypoxia, decreased HIF-1α expression, and reprogrammed TAMs to the M1 phenotype, thereby enhancing antitumor immune responses^[142]^.

#### Nanomaterials for directly repolarizing TAMs

A phospholipid- and PEG-enveloped tannic acid core was developed for the delivery of the CXCR4 antagonist BPRCX807. The nanoagent reprogrammed TAMs toward an M1 phenotype, promoted T cell infiltration, and enhanced the efficacy of PD-1 blockade and whole-cancer-cell vaccines^[56]^. KIRA6, an inhibitor of the hypoxia-activated IRE1-XBP1 pathway, was encapsulated in a reductive nanoemulsion containing α-tocopherol. The nanoemulsion effectively reprogrammed TAMs by inhibiting the hypoxia-activated IRE1-XBP1 axis and reducing oxidative stress, thereby increasing the efficacy of PD-1 antibodies^[57]^. Biocatalytic nanoparticles were synthesized via chelating competition-induced polymerization of a metal-organic framework and dopamine, with the toll-like receptor 7/8 agonist resiquimod and imatinib incorporated, followed by encapsulation with peptides targeting M2 macrophages and Tregs. These nanoparticles reprogrammed M2 macrophages into M1 macrophages, reduced Tregs, and efficiently alleviated hypoxia, thereby enhancing the infiltration of M1 macrophages and T cells^[58]^. Sun *et al.* fabricated a hypoxia-responsive PEGylated Fe-5,5’-azosalicylic acid nanoscale coordination polymer. In the hypoxic milieu, cleavage of the azobenzene bond by azo-reductase released 5-azosalicylic acid and ferric ions, which triggered apoptosis. Meanwhile, 5-azosalicylic acid, as a cyclooxygenase-2 inhibitor, suppressed prostaglandin E_2_ expression, while Fe^3+^ re-educated TAMs toward the M1 phenotype. Collectively, these effects remodeled the immunosuppressive microenvironment to elicit an immune response^[143]^. Biomimetic nanoparticles coated with hybrid cancer-macrophage cytomembranes were designed to load semiconducting polymers, atovaquone, and TMP195 which can repolarize TAMs. These nanoparticles alleviated hypoxia, reprogrammed TAMs toward the M1 phenotype, induced ICD, and converted the TME from a “cold” to a “hot” state^[144]^. In primary tumors, the proportion of CD3^+^CD8^+^ T cells after treatment with the biomimetic nanoparticles plus ultrasound reached 16.8% ± 0.7%, which was significantly higher than that in the control group treated with ultrasound alone (5.9% ± 0.1%). The numbers of CD3^+^CD8^+^ T cells in lymph nodes and distant tumors were also markedly increased.

#### Nanomaterials for depleting TAMs

Direct depletion of TAMs represents another strategy to counteract their role in inducing resistance to immunotherapy. A hemoglobin-poly(ε-caprolactone) conjugate self-assembly was designed to co-deliver doxorubicin and oxygen. The hemoglobin component specifically bound to M2 TAMs via the CD163 receptor, while the loaded doxorubicin effectively killed TAMs. Additionally, oxygen released by hemoglobin alleviated hypoxia and reduced macrophage recruitment^[145]^. *In vivo*, this self-assembled system reduced TAMs from 70.8% ± 7.7% in the control group to 31.3% ± 4.2% (*P* < 0.001), owing to the combined effects of TAM targeting and hypoxia mitigation. Furthermore, liposomes modified with the TAM-targeting peptide M2pep were used to encapsulate zoledronic acid and the sonosensitizer hematoporphyrin monomethyl ether. The combination of sonodynamic therapy and zoledronic acid effectively depleted M2-like TAMs and elicited multi-faceted antitumor immune responses, including relief of tumor hypoxia, increased production of immune-promoting cytokines, and reduced levels of immunosuppressive cytokines^[146]^.

#### Nanomaterials targeting HIF-1α to re-educate TAMs

Liposomes delivering tanespimycin, a potential ICD inducer, were developed. In addition to inducing ICD, tanespimycin acted as a potent Hsp90 inhibitor, downregulating HIF-1α (an Hsp90 client protein), reducing TAMs and MDSCs within the TME, and enhancing the efficacy of immune checkpoint blockade therapy in triple-negative breast cancer^[147]^. In another approach, the hypoxia pathway inhibitor docosahexaenoic acid was conjugated to fucoidan via a cleavable selenylsulfide bond to form micelles, with carfilzomib encapsulated in their hydrophobic core. These micelles induced ICD, suppressed HIF-1α expression, inhibited TAM infiltration and M2 polarization, thereby remodeling the immunosuppressive milieu and enhancing antitumor immune responses^[148]^.

### Nanomaterials inhibiting T cell exhaustion

**Figure 5:**
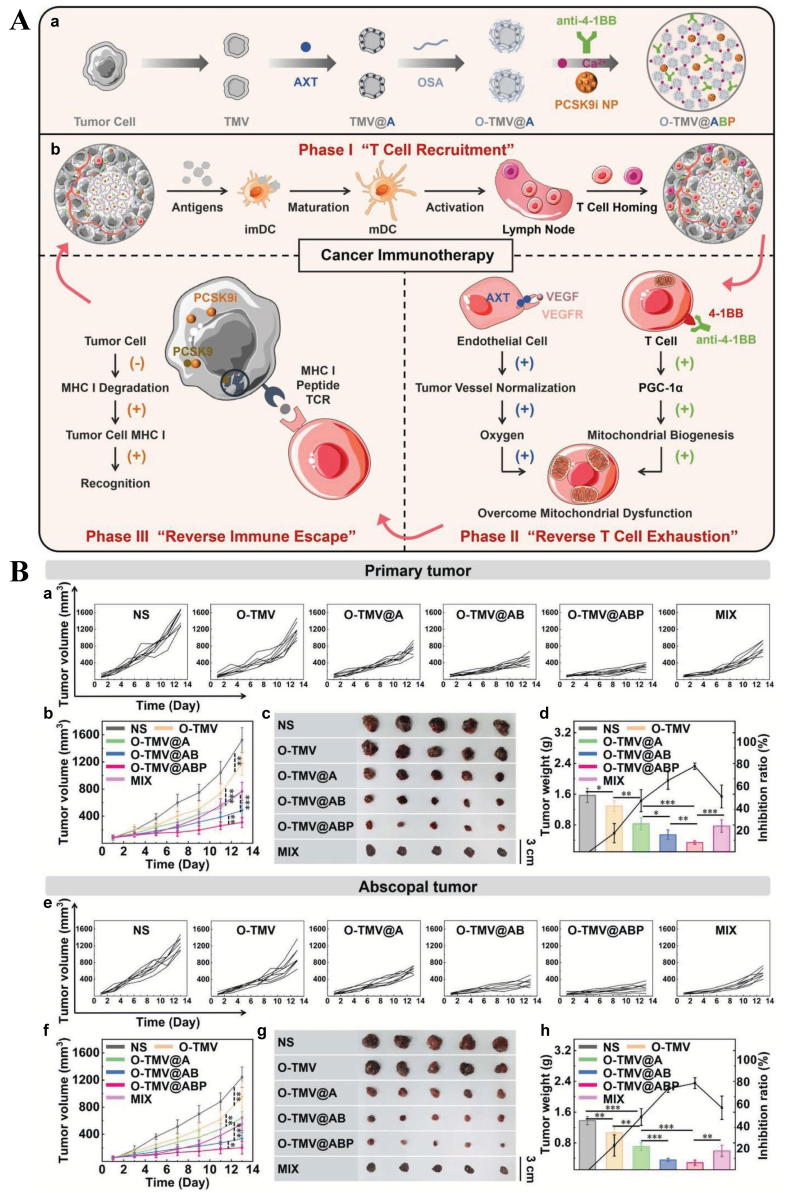
(A) Schematic illustration of the injectable hydrogel and its role in T cell recruitment, reversal of T cell exhaustion, and MHC I-regulated immune escape in cancer immunotherapy; (B) *In vivo* inhibitory effects of the hydrogel on primary tumor and abscopal tumors. ^*^P < 0.05, ^**^P < 0.01, ^***^P < 0.001 [reproduced with permission from Zhang *et al.* (2022)^[150]^. Copyright 2022 John Wiley and Sons]. MHC I: Major histocompatibility complex class I.

Various nanoparticles targeting hypoxia-related pathways and alleviating the hypoxic TME have been developed to inhibit T cell exhaustion. For example, a hydrogel was generated *in situ* within tumors by leveraging oxidized sodium alginate decorated cancer cell membrane vesicles as a gelator. Axitinib was embedded within the lipid bilayer of the membrane, while 4-1BB antibody and the proprotein convertase subtilisin/kexin type 9 inhibitor PF-06446846 were encapsulated in the hydrogel cavities. The cancer cell membrane antigens elicited an immune response, activating and recruiting T cells to the tumor. The released 4-1BB antibody bound to the costimulatory receptor 4-1BB on T cells and enhanced mitochondrial biogenesis, overcoming exhaustion via upregulation of peroxisome proliferator-activated receptor-γ coactivator-1α. Axitinib, a vascular endothelial growth factor receptor (VEGFR) inhibitor, alleviated hypoxia and further prevented T cell exhaustion. Additionally, PF-06446846 enhanced major histocompatibility complex class I (MHC I) expression in carcinoma cells, facilitating their recognition by T cells^[150]^. *In vivo*, the hydrogel achieved a 78% tumor inhibition rate and reduced pulmonary metastatic nodules by 26-fold compared with the control group. The construction of the injectable hydrogel and its mechanism in reversing T cell exhaustion are presented in Figure 5. A stable water-in-oil lipiodol Pickering emulsion stabilized with calcium phosphate nanoparticles was fabricated to encapsulate L-arginine, which modulates T cell metabolism. The emulsion neutralized the acidic TME via calcium phosphate and regulated T cell metabolism through L-arginine, thereby synergistically reversing CD8^+^ T cell exhaustion and tumor immunosuppression^[151]^. In non-small cell lung cancer, hypoxia and lipid rafts in the cell membrane hinder T cell infiltration and impair their function. An albumin-bound fluvastatin nanoformulation simultaneously alleviated hypoxia and disrupted lipid raft integrity, restoring T cell infiltration and enhancing cytotoxic T cell function, ultimately improving the efficacy of anti-PD-1 antibody therapy^[152]^.

Manganese dioxide-albumin nanoparticles were used as drug carriers to load buformin (an inhibitor of mitochondria-associated oxidative phosphorylation) and methylene blue (a photodynamic therapy agent with PD-1 inhibition activity) via electrostatic absorption. Hypoxia was alleviated by inhibiting O_2_ consumption with buformin and generating O_2_ through MnO_2_ activity, thereby enhancing photodynamic therapy. Furthermore, the manganese dioxide-albumin complex strengthened ICD, inhibited the PD-1/PD-L1 axis, and relieved T cell exhaustion^[153]^. Cancer cell membrane - encapsulated manganese oxide nanozymes with multienzyme-mimicking activity exhibited peroxidase- and oxidase-like functions and induced ICD. The released Mn^2+^ promoted dendritic cell maturation and TAM reprogramming, while catalase-like activity relieved tumor hypoxia. Collectively, these effects reversed the immunosuppressive TME and significantly increased the proportions of CD8^+^ cytotoxic T lymphocytes and CD4^+^ T cells within tumors. Combination therapy with the manganese oxide nanozyme and PD-1 antibody further enhanced T cell-mediated antitumor immunity^[154]^. Liposomes were also engineered to co-deliver metformin, which downregulates PD-L1 expression via AMP-activated protein kinase-mediated ER-associated protein degradation, and IR775, a photodynamic therapy agent. These liposomes alleviated tumor hypoxia to boost ROS production, reduced PD-L1 expression, and reversed T cell exhaustion^[155]^.

CaO_2_ nanoparticles encapsulated with EL4 cell membranes effectively rescued T cells from exhaustion by increasing glucose availability for cytotoxic lymphocytes and decreasing lactic acid accumulation through Ca^2+^-mediated blockade of glycolysis. They also alleviated hypoxia, scavenged TGF-β1, and blocked PD-L1 via cell membrane receptors^[156]^. *In vivo*, treatment with CaO_2_ nanoparticles and 2-deoxyglucose increased tumor-infiltrating IFN-γ^+^ CD8^+^ T cells by 6.1-fold, demonstrating effective rescue of CD8^+^ T cells from exhaustion in the immunosuppressive TME. Remarkably, this combination also reduced MDSCs and Tregs by 30.6% and 28.6%, respectively.

### Nanomaterials retarding immune escape

**Figure 6:**
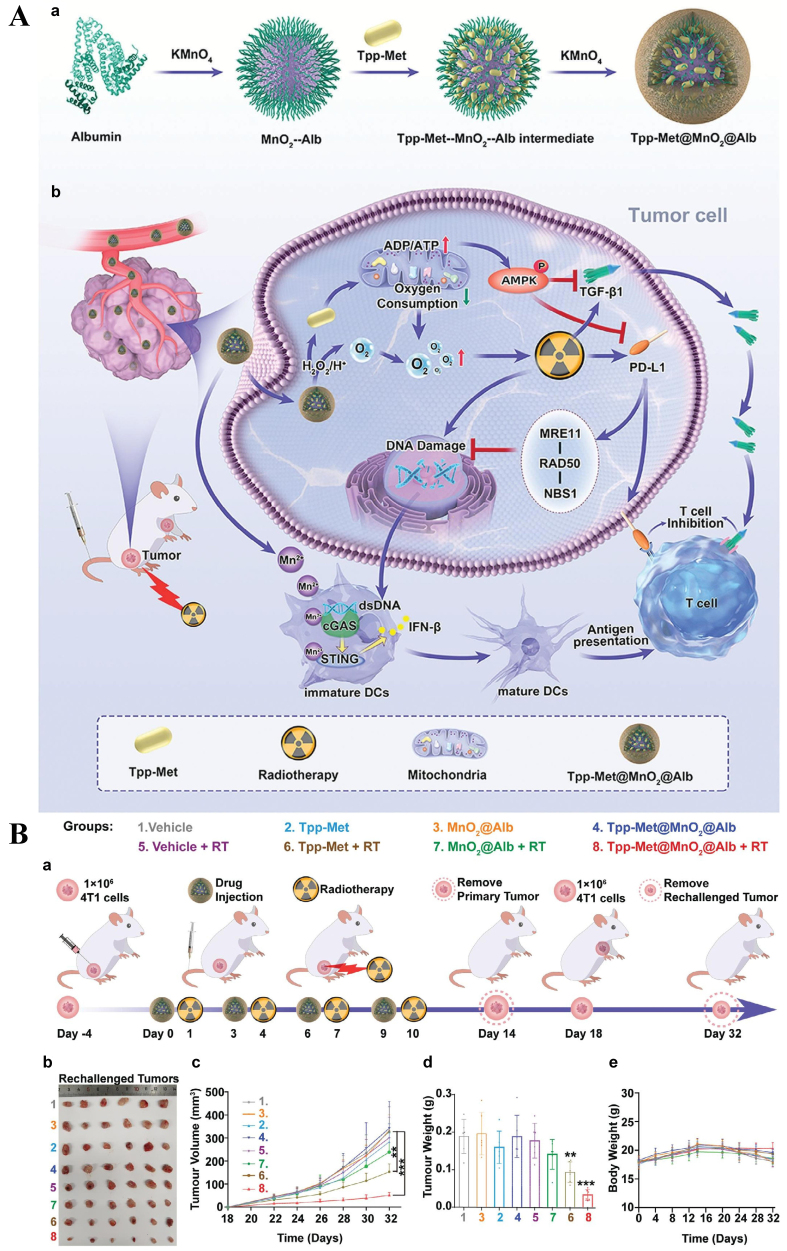
(A) Albumin-templated manganese dioxide nanoparticles loaded with triphenylphosphine-derived metformin via positive and negative adsorption to form a hybrid nanoadjuvant; (B) These nanoparticles synergized with radiotherapy to elicit antitumor immunological memory. ^**^P < 0.01, ^***^P < 0.001 [reproduced with permission from Yi *et al.* (2024)^[157]^. Copyright 2024 John Wiley and Sons].

The PD-1/PD-L1 axis is a key pathway mediating immune evasion, and numerous nanoformulations have been developed to alleviate hypoxia or inhibit PD-1/PD-L1-mediated immune escape. As depicted in Figure 6, a hybrid nanoadjuvant was fabricated by loading triphenylphosphine-derived metformin - an agent that decreases oxygen consumption by actively targeting mitochondria and inhibiting complex I of the respiratory chain - into albumin-templated manganese dioxide nanoparticles through positive and negative adsorption. Tumor hypoxia was alleviated by increased O_2_ production catalyzed by MnO_2_ and decreased O_2_ consumption induced by metformin-mediated mitochondrial inhibition. In addition, metformin suppressed TGF-β secretion and reduced membrane-localized PD-L1 expression, thereby reversing the immunosuppressive microenvironment and activating T cells^[157]^. Liposomes co-loaded with metformin, catalase, and hematoporphyrin monomethyl ether were designed to relieve hypoxia and enhance the efficacy of photoimmunotherapy in “cold” tumors. Hypoxia was reversed through H_2_O_2_ decomposition catalyzed by catalase, along with reduced O_2_ consumption via metformin. Furthermore, metformin downregulated PD-L1 expression in tumor cells, markedly enhancing T cell cytotoxicity^[158]^. Triphenylphosphine cations targeting mitochondria were conjugated to the antineoplastic agent lonidamine, and the conjugate was encapsulated into liposomes. These liposomes reversed hypoxia, downregulated PD-L1 expression by activating AMP-activated protein kinase, and sensitized tumors to radiotherapy^[159]^. *In vivo*, the combination of these liposomes with radiotherapy almost completely suppressed tumor growth, achieving inhibition rates of 79.6% ± 5.2% by day 14. A mitochondria-targeted heptamethine cyanine photodynamic therapy dye (MHI) was conjugated with tamoxifen, an inhibitor of mitochondrial complex I, and the conjugate further self-assembled with albumin. The resulting nanoparticles effectively alleviated tumor hypoxia, inhibited PD-L1 expression, and enhanced the efficacy of photodynamic immunotherapy by promoting T cell infiltration^[160]^.

In addition to strategies that reduce oxygen consumption to mitigate hypoxia, other nanoagents have been developed to generate oxygen. Chen *et al.* fabricated multienzyme-mimetic alloy nanosheets composed of palladium and iron. These nanosheets exhibited peroxidase- and catalase-like activities, as well as the ability to induce ferroptosis. They counteracted hypoxia through catalase-like O_2_ generation, resulting in decreased HIF-1α expression, reduced infiltration of M2-like macrophages and Tregs, and suppressed PD-L1 expression. Moreover, the nanosheets possessed second near-infrared phototherapy and photoacoustic imaging capabilities, and combined with their ferroptosis induction and TME-modulating properties, they synergized effectively with anti-PD-L1 treatment^[161]^. A multifunctional nanocomposite consisting of defect-rich tungsten trioxide (WO_3-x_) and ferrocene-folic acid was synthesized via sequential reactions of ferrocenyl chloride with folate and WO_3-x_. This composite triggered ICD through synergistic photothermal and chemodynamic therapy, while simultaneously decomposing H_2_O_2_ to O_2_ via the Fenton reaction, thereby reducing hypoxia-induced PD-L1 expression^[162]^.

A liposomal nanovehicle co-loaded with copper oleate, a Fenton catalyst, and the HIF-1 inhibitor acriflavine downregulated PD-L1 expression by inhibiting HIF-1, while copper oleate-mediated chemodynamic therapy was enhanced by suppression of the HIF-1 signaling pathway^[163]^. Monomethoxy PEG-poly(lactic-co-glycolic acid) nanoparticles encapsulating the hypoxia-activated prodrug evofosfamide (TH-302), a 2-nitroimidazole derivative that releases bromo-isophosphoramide mustard, effectively ameliorated tumor hypoxia, reduced HIF-1α and PD-L1 expression, and facilitated infiltration of CD8^+^ T cells, thereby potentiating anti-PD-1 therapy^[164]^. Combined nanoparticles co-loaded with TH-302 and an anti-PD-L1 antibody significantly reduced tumor volume (*P* < 0.01 *vs.* anti-PD-1 group) and tumor weight (*P* < 0.001 *vs.* anti-PD-1 group). Clustered regularly interspaced short palindromic repeats/Cas9 (CRISPR/Cas9) technology has also emerged as a promising approach for stably altering gene expression. Silica-decorated silver sulfide quantum dots, which emit in the second near-infrared window, were engineered to deliver CRISPR/Cas9 ribonucleoproteins via a hypoxia-responsive azo bond. The quantum dots were encapsulated by amphiphilic tirapazamine-modified hyaluronic acid polymers, which were further cross-linked with disulfide bonds. Under hypoxic conditions, activation of the tirapazamine prodrug and spatiotemporal release of the CRISPR/Cas9 ribonucleoprotein were achieved. This strategy significantly alleviated hypoxia by depleting HIF-1α and activating tirapazamine, thereby disrupting PD-1/PD-L1 signaling and enhancing T cell-mediated antitumor immunity^[165]^.

### Nanoparticles for tumor vascular normalization

**Figure 7:**
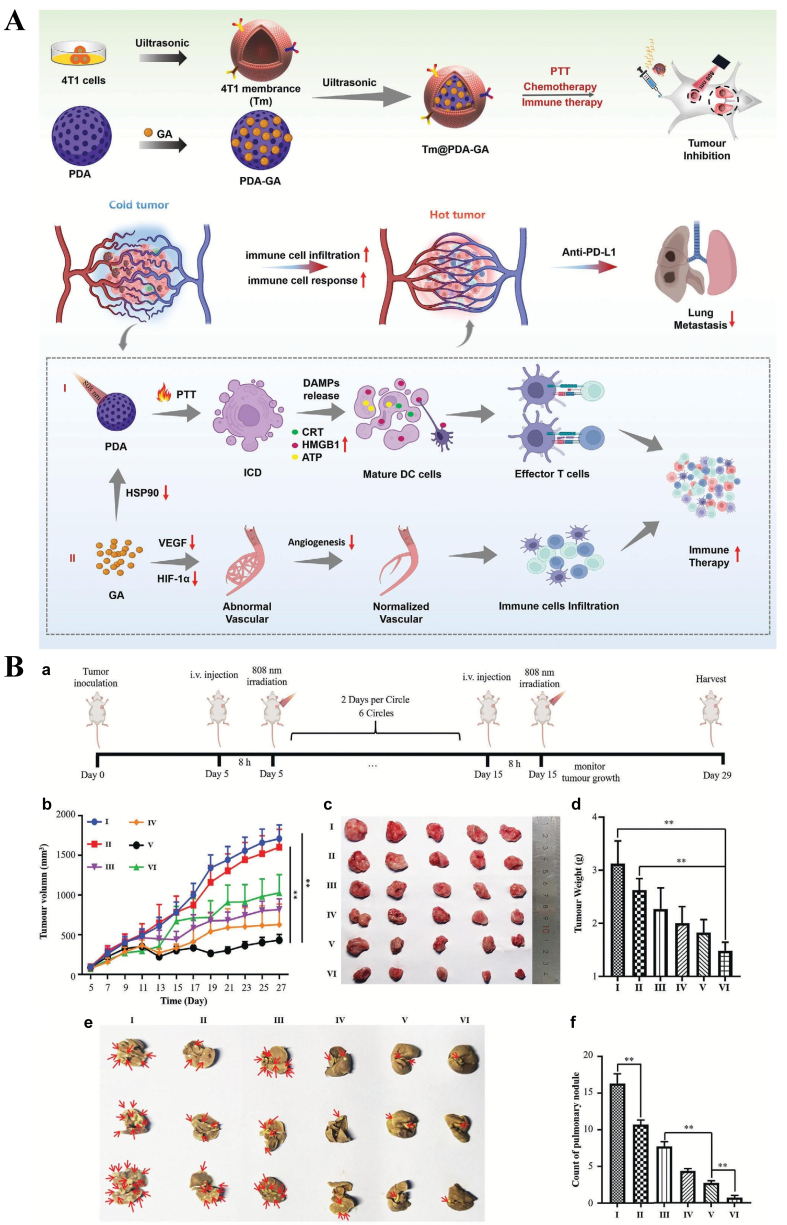
(A) Design and features of 4T1 cytomembrane-camouflaged polydopamine nanoparticles for delivery of gambogic acid; (B) *In vivo* antitumor and antimetastatic efficacy. ^**^P < 0.01 [reproduced with permission from Lan *et al.* (2024)^[167]^. Copyright 2024 John Wiley and Sons].

Nanoparticles for tumor vascular normalization have emerged as a rational strategy to alleviate tumor hypoxia and overcome resistance to immunotherapy. A unique peptide amphiphile was developed by incorporating antiangiogenic secreted protein acidic and rich in cysteine (SPARC) FSEC peptide and PD-L1-inhibiting ^D^PPA peptide sequences, linked via a legumain-cleavable amino acid sequence, and further self-assembled into nanostructures through grafting with hydrophobic octadecylamine tails. The antiangiogenic peptide promoted vascular normalization, thereby enhancing intratumoral infiltration of CD8^+^ T cells and NK cells while reducing tumor hypoxia. At the same time, inhibition of PD-L1 relieved immune suppression and enabled an effective antitumor immune response^[166]^. Polydopamine, which can trigger photothermal therapy and induce ICD, was employed to deliver gambogic acid and further camouflaged with 4T1 cell membranes to construct a biomimetic immunostimulatory nanomodulator. Gambogic acid, released in response to the acidic TME, suppressed heat shock proteins to synergize chemo-photothermal therapy with ICD. In addition, gambogic acid inhibited HIF-1α and VEGF, leading to tumor vascular normalization, reduced hypoxia stress, and improved immune cell infiltration. This synergistic approach transformed a “cold” tumor into a “hot” tumor and enhanced the efficacy of anti-PD-L1 therapy [Figure 7]^[167]^. The combined biomimetic nanomodulator, light irradiation, and anti-PD-L1 treatment achieved an impressive tumor inhibition rate of 77.29% (*P* < 0.01 *vs.* the anti-PD-L1 group), and the number of lung metastatic foci was reduced to 0.67 ± 0.33, significantly lower than in other groups (*P* < 0.01).

Lenvatinib, axitinib, and other tyrosine kinase inhibitors with antiangiogenic activity have also been applied to normalize tumor vasculature and mitigate hypoxic stress. iRGD (CRGDKGPD)-decorated, pH-responsive liposomes co-encapsulating lenvatinib and the small-molecule PD-1/PD-L1 inhibitor BMS achieved vascular normalization, reduced Tregs and MDSCs, promoted CD8^+^ T cell infiltration, and upregulated PD-L1 expression on cancer cells^[168]^. Similarly, a novel nanoparticle composed of a biodegradable second near-infrared fluorescent pseudo-conjugate polymer and lenvatinib alleviated hypoxia through vascular normalization, thereby improving photodynamic therapy efficacy, T cell infiltration, and dendritic cell maturation^[169]^. Co-assembly of lenvatinib, adriamycin, Fe^3+^ ions, and the natural polyphenol epigallocatechin-3-gallate normalized tumor vasculature, enhanced T cell infiltration, and reduced Tregs and PD-L1 expression on tumor cells^[170]^. A human serum albumin (HSA)-based self-delivery nanoagent was also constructed through co-assembly of verteporfin, axitinib, and celecoxib. Axitinib-mediated vascular normalization reduced tumor hypoxia and reversed VEGF-driven immunosuppression, thereby promoting infiltration of effector immune cells^[171]^. Another HSA-based assembly of axitinib, the photosensitizer chlorin e6, and the IDO inhibitor dextro-1-methyl tryptophan simultaneously enhanced photodynamic therapy, alleviated hypoxia, normalized vasculature, and boosted immune cell infiltration, collectively strengthening immunotherapy efficacy^[172]^. An injectable thermosensitive PLGA-PEG-PLGA copolymer hydrogel co-delivering the vascular-disruptive agent combretastatin A4 disodium phosphate and epirubicin promoted CD8^+^ T cell infiltration and dendritic cell maturation, while reducing MDSCs and Tregs^[173]^. This hydrogel exerted vascular disrupting effects and achieved a tumor inhibition rate of 92% *in vivo* compared with the saline group. In another strategy, a self-assembled conjugate of the photodynamic dye MHI148 with bovine serum albumin and sorafenib enabled cascade two-step reoxygenation and immune re-sensitization. Sorafenib decreased oxygen consumption by inhibiting mitochondrial respiration while also increasing oxygen supply through vascular normalization, thereby enhancing T cell infiltration and reducing PD-L1 expression^[174]^. Finally, a polymersome-based platform for delivering the cyclic dinucleotide STING agonist not only promoted vascular normalization and mitigated hypoxia but also upregulated T cell adhesion molecules, enhanced T cell infiltration, proliferation, and function, and amplified the efficacy of immune checkpoint inhibitors and adoptive T cell treatment^[175]^.

## CONCLUSION AND PERSPECTIVES

Immunotherapy has emerged as a crucial therapeutic strategy for cancer, complementing chemotherapy, surgery, radiotherapy, and targeted therapy, and patients have benefited from a wide range of immunotherapeutic approaches. Despite its great potential, cancer immunotherapy still faces significant challenges, with low response rates remaining a major hurdle. The HIF-1α signaling pathway has been shown to play a central role in resistance to immunotherapy. Recent advances in nanotechnology have enabled the reversal of immunotherapy resistance through nanomaterials targeting the HIF-1α axis. However, several issues in both basic research and, more importantly, clinical application remain unresolved.

The precise mechanisms and signaling pathways underlying HIF-1α-mediated immunotherapy resistance are not yet fully understood, and further in-depth molecular studies are required. The metabolism and long-term safety of nanomaterials - particularly inorganic nanoparticles - remain insufficiently characterized, necessitating additional investigation. Potential safety risks associated with long-term or repeated administration of nanoparticles targeting the HIF-1α pathway, especially in the context of chronic immunotherapy regimens, must be carefully evaluated. Minimizing off-target effects and the accumulation of nanoparticles in non-target organs is essential for safety, as is the prevention of immune overactivation. Thus, the optimization of material formulations and the development of more biocompatible nanomaterials are ongoing priorities.

Tumor hypoxia is characterized by heterogeneity across tumor types and clinical stages, as well as spatial heterogeneity within individual tumors^[17]^. Therefore, the design of nanoparticles targeting the hypoxia axis with diverse therapeutic strategies should be informed by further fundamental studies in oncology, pathology, and clinical staging. Moreover, factors influencing the response to immunotherapy are highly complex and extend beyond tumor hypoxia alone. Integrated strategies to overcome resistance and achieve personalized treatment represent a promising research direction.

When designing nanomaterials targeting the hypoxia signaling pathway, druggability must also be carefully considered. The pharmaceutical industry generally follows the principle of “keep it simple, stupid”, and complex manufacturing processes and standardization challenges hinder the scale-up of nanodrugs from laboratory to industry. The pharmacokinetics and *in vivo* behavior of nanomedicines remain poorly defined, particularly in humans, and urgent, in-depth investigations are required. The complexity of nanoagents has also prompted regulatory agencies to advocate for more stringent standardization protocols, comprehensive toxicity profiling, and rigorous efficacy evaluations to ensure safety, potency, and reproducibility.

As of 2025, ClinicalTrials.gov lists no ongoing or completed clinical trials investigating hypoxia and nanoparticles. This absence is likely due to the challenges of large-scale manufacturing and safety concerns regarding nanoparticles. Robust preclinical data on safety and efficacy in large-scale animal tumor xenograft models are essential for clinical translation. To improve clinical prospects, simple and biocompatible nanosystems targeting the hypoxia pathway should be developed. Well-controlled clinical trials are urgently needed to define the limitations and therapeutic potential of nanomaterials targeting hypoxia for overcoming immunotherapy resistance.

In conclusion, cancer immunotherapy still faces major challenges, yet strategies targeting the hypoxia signaling pathway to overcome resistance have shown encouraging progress in research. We anticipate that continued basic research on hypoxia and immunotherapy resistance will enable the development of simpler, safer nanocarriers that can be translated into clinical practice for personalized treatment, ultimately allowing more patients to benefit from immunotherapy.

## DECLARATIONS

### Copyright

© The Author(s) 2025.
